# Significant improvement of bone marrow-derived MSC expansion from MDS patients by defined xeno-free medium

**DOI:** 10.1186/s13287-023-03386-5

**Published:** 2023-06-07

**Authors:** Eva Altrock, Carla Sens-Albert, Franziska Hofmann, Vladimir Riabov, Nanni Schmitt, Qingyu Xu, Johann-Christoph Jann, Felicitas Rapp, Laurenz Steiner, Alexander Streuer, Verena Nowak, Julia Obländer, Nadine Weimer, Iris Palme, Melda Göl, Ali Darwich, Patrick Wuchter, Georgia Metzgeroth, Mohamad Jawhar, Wolf-Karsten Hofmann, Daniel Nowak

**Affiliations:** 1 Department of Hematology and Oncology, Medical Faculty Mannheim, Heidelberg University, Theodor-Kutzer-Ufer 1-3, 68167 Mannheim, Germany; 2 Department of Orthopedics and Traumatology, Medical Faculty Mannheim, Heidelberg University, Theodor-Kutzer-Ufer 1-3, 68167 Mannheim, Germany; 3 Institute of Transfusion Medicine and Immunology, Medical Faculty Mannheim, Heidelberg University, German Red Cross Blood Service Baden-Württemberg-Hessen, Friedrich-Ebert-Str. 107, 68167 Mannheim, Germany

**Keywords:** Bone marrow-derived mesenchymal stromal cells, Myelodysplastic Neoplasms, Xeno-free expansion

## Abstract

**Background:**

Robust and reliable in vitro and in vivo models of primary cells are necessary to study the pathomechanisms of Myelodysplastic Neoplasms (MDS) and identify novel therapeutic strategies. MDS-derived hematopoietic stem and progenitor cells (HSPCs) are reliant on the support of bone marrow (BM) derived mesenchymal stroma cells (MSCs). Therefore, isolation and expansion of MCSs are essential for successfully modeling this disease. For the clinical use of healthy MSCs isolated from human BM, umbilical cord blood or adipose tissue, several studies showed that xeno-free (XF) culture conditions resulted in superior growth kinetics compared to MSCs cultured in the presence of fetal bovine serum (FBS). In this present study, we investigate, whether the replacement of a commercially available MSC expansion medium containing FBS with a XF medium is beneficial for the expansion of MSCs derived from BM of MDS patients which are often difficult to cultivate.

**Methods:**

MSCs isolated from BM of MDS patients were cultured and expanded in MSC expansion medium with FBS or XF supplement. Subsequently, the impact of culture media on growth kinetics, morphology, immunophenotype, clonogenic potential, differentiation capacity, gene expression profiles and ability to engraft in immunodeficient mouse models was evaluated.

**Results:**

Significant higher cell numbers with an increase in clonogenic potential were observed during culture of MDS MSCs with XF medium compared to medium containing FBS. Differential gene expression showed an increase in transcripts associated with MSC stemness after expansion with XF. Furthermore, immunophenotypes of the MSCs and their ability to differentiate into osteoblasts, adipocytes or chondroblasts remained stable. MSCs expanded with XF media were similarly supportive for creating MDS xenografts in vivo as MSCs expanded with FBS.

**Conclusion:**

Our data indicate that with XF media, higher cell numbers of MDS MSCs can be obtained with overall improved characteristics in in vitro and in vivo experimental models.

**Supplementary Information:**

The online version contains supplementary material available at 10.1186/s13287-023-03386-5.

## Background

The term MSCs describe a cell population of multipotent stem/progenitor cells commonly referred to as mesenchymal stem cells, multipotential stromal cells or mesenchymal stromal cells [1]. MSCs have become widely used for clinical studies in the field of tissue engineering and stem cell therapy for the treatment of bone diseases, cartilage repair, myocardial infarction and graft versus host disease [1, 2]. Originally, MSCs were isolated and characterized from bone marrow but nowadays they can be derived from various postnatal tissues, such as adipose tissue, dental pulp, umbilical cord and cord blood (UCB), amniotic fluid and limbal tissue [3]. The use of MSCs requires ex vivo expansion to reach appropriate cell numbers. Although there are common criteria for the definition of MSCs by the International Society for Cellular Therapy (ISCT) [4] such as plastic adherence, defined expression of CD73, CD90 and CD105 with lack of the expression of hematopoietic markers and differentiation potential into adipocytes, osteoblasts and chondroblasts in vitro, MSCs may differ in secretome and /or transcriptome profile depending on the donor and the site of collection [5].

WHO classification of hematologic neoplasia was updated in 2022 and changed the term for MDS from Myelodysplastic Syndromes to Myelodysplastic Neoplasms [6, 7]. MDS are a heterogeneous group of malignant hematopoietic stem cell (HSC) disorders that predominantly affect older individuals [8–12]. The prognostic classification of MDS is based on the international scoring systems IPSS (International Prognostic Scoring System) / IPSS-R [13, 14] and the recently established IPSS-M (Molecular International Prognostic Scoring System)[15]. For a long time, disease progression and initiation have been considered to be exclusively driven by hematopoietic cells. However, several reports show a more active role of the bone marrow (BM) niche suggesting a contribution of the BM microenvironment to disease pathogenesis [16–18]. Therefore, the use of patient-derived MSCs for modeling MDS seems to be essential [19, 20]. One goal of our group is the improvement of our MDS patient-derived xenografts (PDX) to study the pathomechanisms of MDS and to find new therapeutic strategies. It has been demonstrated that MSCs in MDS are intrinsically pathological and that senescence is increased by a continuous decline in proliferation [21–23]. Ex vivo expanded MSCs from MDS patients display altered differentiation characteristics, transcriptional abnormalities and a reduced ability to support HSPCs [17, 23, 24]. However, in contrast to MDS HSPCs, MDS MSCs do not carry disease-specific clonal mutations in vivo [25]. The isolation and expansion of MDS MSCs from patient BM are challenging. For the establishment of PDX models of MDS our group previously established the isolation of MSCs through plastic adherence from the BM of MDS patients using a culture medium containing fetal bovine serum (FBS). In most culture systems, FBS is an essential component for cell growth and maintenance. However, animal sera are a potent risk of microbial and viral contamination and immunological reactions [26]. The composition of FBS is unknown and differs from batch to batch. Furthermore, there are ethical concerns using FBS and in terms of the 3Rs (Refinement, Reduction and Replacement of animal experiments in research) so that serum-free alternatives would be favorable [27]. For clinical use, several studies showed that xeno-free (XF) culture conditions resulted in superior growth kinetics of MSCs isolated from human BM, UCB or adipose tissue compared to cultivation with FBS [3, 5, 26].

In the current study, we investigated, whether the replacement of a commercially available MSC growth medium containing FBS with a XF medium would be beneficial for the expansion of MSC derived from BM of MDS patients. We therefore compared the growth kinetics of *n* = 12 MDS MSCs cultured in MSC expansion medium with FBS or XF supplement. In addition, the ability to form CFU-Fs (Colony-forming unit fibroblast), differentiation potential and gene expression was assessed. Our data indicate that growth kinetics were affected by the medium formulation with a higher proliferation rate and average number of CFU-Fs using XF culture media. These cells fulfilled the minimal criteria for MSCs and supported human engraftment of MDS BM cells similar as cells obtained after culture with FBS. Taken together our data show an improvement of the culture of bone marrow-derived MSCs from MDS patients to gain more cells for xenotransplantation.

## Methods

### Patient samples and healthy controls

We included primary BM samples from diagnostic BM aspirations of *n* = 12 patients diagnosed with MDS. Patient characteristics are depicted in Table 1. As healthy controls, primary BM from young donors (*n* = 4, age ranges from 22 to 23 years) or from elderly donors (*n* = 11, age ranges from 64 to 79 years) after hip joint endoprosthetic surgery at the Department of Orthopedics and Traumatology of the Medical Faculty Mannheim, Heidelberg University, Germany, was used. The use of primary material followed written informed consent of patients and approval by the Institutional Review Board of the Medical Faculty Mannheim, University of Heidelberg, Germany, in accordance with the Declaration of Helsinki. Samples were processed by Ficoll density gradient centrifugation to enrich for mononuclear cells (MNCs).

**Table 1:** MDS patient characteristics of the material used for in vitro experiments

Patient ID	WHO 2016	IPSS	Agerange	Sex	Karyotype	Tracking lesions
P01	MDS-MLD	low	60–65	m	46,XY[20]	*TET2*
P02	MDS-EB1	int-1	60–65	m	46,XY[20]	*SF3B1*
P03	MDS-MLD	int-2	70–75	m	46,XY[20]	Not detected
P04	MDS-EB1	int-2	60–65	m	46,XY, + 1.der(1;16)(q10;p10)[18/20]	*RUNX1, IDH1, IDH2, SRSF2*
P05	MDS-EB2	high	60–65	m	45,X, -Y [11]; 45,X,-Y,del(9)(q21q32) [5]; 45,X,-Y,del(9)(q13q34)[4]	*KRAS*
P06	MDS-EB2	int-2	40–45	f	45,XX,-7[15]	*IDH2*
P07	MDS-MLD	int-1	70–75	m	46,XY[20]	Not detected
P08	MDS-RS-MLD	low	70–75	m	46,XY[20]	*SF3B1*
P09	MDS-MLD	int-1	80–85	m	46,XY[20]	Not detected
P10	MDS-MLD	int-1	80–85	f	46, XX[25]	*ASXL1, DNMT3A*
P11	MDS-EB1	int-1	55–60	f	46,XX[20]	*TP53, SF3B1*
P04_2	MDS-EB1	int-2	60–65	m	46,XY, + 1.der(1;16)(q10;p10)[18/20]	*RUNX1, IDH1, IDH2, SRSF2*

The table displays the WHO classification 2016, IPSS, age, sex and karyotype of the MDS patients whose MSCs were used for experiments

*MDS* Myelodysplastic Neoplasms, *IPSS* MDS risk score according to international prognostic scoring system, *int* Intermediate, *m* Male, *f* Female

### Isolation and culture of bone marrow-derived MSCs

MSCs were isolated as described previously [19]. Briefly, MNCs from BM aspirations or hipbone were seeded into T25 cell culture flasks and MSCs were enriched by their plastic adherence. MSCs were cultured using StemMACS human MSC Expansion Media (FBS) with weekly medium change. After reaching more than 9 × 10^5^ MSCs, two-thirds were stored viably in liquid nitrogen. To compare growth kinetics between healthy and MDS MSCs 330 MSCs/cm^2^ freshly thawed samples were washed two-times in the target medium and were cultured with StemMACS human MSC Expansion Media (FBS) or StemMACS human MSC Expansion Media XF (Miltenyi Biotec, Bergisch Gladbach, Germany) for 12 days. Cell numbers were counted with a Vi-CELL cell viability analyzer (Beckman Coulter, Krefeld, Germany) every 2–3 days. Representative images were captured with an inverted Leica DMi1 microscope and cell area was measured for *n* = 7 healthy samples and *n* = 7 MDS samples using ImageJ (Wayne Rasband, National Institutes of Health).

### Colony forming unit-fibroblast assay

To test the colony forming capacity of MSCs, the cells were plated after thawing from passage 1 at a density of 100 cells per well in a 6-well plate and incubated for 10–14 days. Colonies were visualized with Giemsa staining. Only clones consisting of more than 50 cells were included and manually counted. The assay for each sample was carried out in triplicate.

### Tri-lineage differentiation of MSCs

The Mesenchymal and Tissue Stem Cell Committee of the International Society for Cellular Therapy proposes minimal criteria to define human MSC. One of these criteria is that MSCs must be able to differentiate to osteoblasts, adipocytes and chondroblasts in vitro [4]. Therefore, we determined the differentiation ability of our MSCs after culture in MSC Expansion Media (FBS) or MSC Expansion Media XF (Miltenyi Biotec, Bergisch Gladbach, Germany). Osteogenic and adipogenic differentiation was initiated in confluent cultures of MSCs (50,000 cells per well in a 96-well plate) using α-MEM (Thermo Fisher Scientific, Schwerte, Germany) containing 10% FBS and 1% Penicillin–Streptomycin (10,000 U/mL, Thermo Fisher Scientific, Schwerte, Germany). For osteogenic differentiation, the medium was supplemented with 5 mM β-Glycerophosphate, 50 μg/ml Ascorbic acid and 10 nM Dexamethasone and changed every 2–3 days. Mineralized deposits were visualized by von Kossa staining after 14–21 days. Cells were fixed with ice-cold 95% Ethanol and 5% Isopropanol at 4 °C for 1 h and exposed to 25 mM silver nitrate under ultraviolet light for 10–20 min. Nodules stained with von Kossa stain after 14–21 days were quantified using ImageJ (Wayne Rasband, National Institutes of Health) [28].

For adipogenic differentiation, on day 1 and 3 medium was supplemented with 3-Isobutyl-1-methylxanthine (IBMX; 0.0115 g/ml in 0.5 M KOH; 1:100), 1 μg/ml Insulin and 1 μM Dexamethasone. On day 5 and 7, the medium was supplemented with 1 μg/ml Insulin, and from day 9, the basic medium was used without supplements. At day 14–21, the presence of lipid droplets was assessed by Oil Red O stain. Cells were fixed with 4% paraformaldehyde for 30 min at room temperature and incubated for 1 h with Oil Red O staining solution (60 ml Oil Red O stock solution (0.5 g Oil Red O + 100 ml 2-Propanol) + 40 ml distilled water filtered with Whatman paper). Chondrogenic differentiation was induced with 5 × 10^5^ MSCs per 96-round-bottom well with MesenCult™-ACF Chondrogenic Differentiation Medium (Stemcell Technologies, Vancouver, Canada). After 21 days Toluidine Blue staining was performed to visualize proteoglycans. For quantification, the stained area was measured using ImageJ (Wayne Rasband, National Institutes of Health).

### Flow cytometry and cell sorting

Flow cytometry analysis was performed on a BD FACSCelesta (BD Biosciences, Heidelberg, Germany) and data were analyzed using FlowJo V10 software. Viably frozen or cultured MSCs were stained 30 min at 4 °C with the following antibodies: Anti-hCD45-FITC (clone HI30, BD Biosciences, Heidelberg, Germany), Human Lineage Cocktail 4 (lin4)-FITC (BD Biosciences, Heidelberg, Germany), anti-hCD34-FITC (clone 561, Biolegend, London, UK), anti-hCD73-APC (clone AD2, Biolegend, London, UK), anti-hCD90-PerCP-Cy5.5 (clone 5E10, BD Biosciences, Heidelberg, Germany), anti-hCD271-BV786 (clone C40-1457, Thermo Fisher Scientific, Schwerte, Germany), anti-hCD146-PE (clone P1H12, Biolegend, London, UK) or anti-hCD105-PE (clone 43A3, BD Biosciences, Heidelberg, Germany). For discrimination of live and dead cells, SYTOX™ Blue Dead Cell Stain (Thermo Fisher Scientific, Schwerte, Germany) was added at 1:2000 in FACS buffer after antibody staining.

### RT^2^ profiler PCR array

Human Mesenchymal Stem Cell RT^2^ Profiler PCR Array (PAHS-082, SABiosciences, Qiagen, Hilden, Germany) was used to evaluate the expression of 84 specific genes related to stemness (pluripotency), MSCs, and cell differentiation (osteogenesis, adipogenesis, chondrogenesis, myogenesis, and tenogenesis) [29]. MSCs from four different patients were used after five days of culture with StemMACS human MSC Expansion Media (FBS) or MSC Expansion Media XF (Miltenyi Biotec, Bergisch Gladbach, Germany). The total RNA was isolated from 10^3^ to 10^5^ cells using the RNeasy Micro Kit (Qiagen, Hilden, Germany) according to the manufacturer's instructions. cDNA was synthesized from 500 ng of the total RNA using the RT^2^ First Strand Kit (Qiagen), which includes the additional removal of genomic DNA from the RNA sample and a specific control of reverse transcription. The samples were analyzed using the RT^2^ Profiler PCR Array. Altogether, 84 different genes were simultaneously amplified in the sample. A melting curve analysis was performed to verify that the product consisted of a single amplicon. PCR arrays were performed in 96-well plates on a LightCycler 480 II instrument (Roche Applied Science). The data were analyzed via Roche LightCycler 480 software, and the *C*_*t*_ values were extracted for each gene. The thresholds and baselines were set according to the manufacturer's instructions (SABiosciences, Qiagen). The fold change in gene expression (compared to cells expanded with FBS) was calculated using the ΔΔ*C*_*t*_ method. A fold change > 2 with *p* < 0.05 in gene expression (compared to cells expanded with FBS) was considered as the up- or downregulation of a specific gene expression.

### Mouse experiments

MDS xenograft experiments for the comparison of FBS versus XF medium cultured MSCs were either performed de novo (*n* = 11 for FBS and *n* = 5 for XF) or additionally inferred from data acquired from a previously published study by Schmitt N et al. [30], in which XF cultured MDS MSCs were used. All animal experiments were approved by the regional state authorities in Karlsruhe, Germany, and were performed using immunodeficient female NOD.Cg-Prkdscid Il2rgtm1Wjl/Szj (NSG) mice (The Jackson Laboratory, JAX stock #005557) [31, 32]. Eight-week-old female NSG mice were transplanted with a previously published standard protocol consisting of intrafemoral (IF) co-injection of CD34 + HSPCs and MSCs [19]. Briefly, approximately 2 × 10^5^ patient-derived CD34 + HSPCs and approximately 1 × 10^6^ in vitro expanded MSCs from the same patient were combined and injected into the femur after conditioning with 25 mg/kg body weight (BW) Busulfan intraperitoneally 48 and 24 h before. Final analysis of engraftment was investigated 32 weeks after transplantation.

### Statistical analysis

Data are presented as mean ± SD. Data were analyzed using Prism 8 (GraphPad Software, La Jolla, CA) using one-way analysis of variance (ANOVA) Kruskal–Wallis test. *P* values were considered significant at values less than 0.05 (ns: not significant, statistically significant *p < 0.05, **p < 0.01, *** < 0.001, ****p < 0.0001).

## Results

### Defined XF medium increases cell numbers of healthy and MDS-derived MSCs during in vitro culture

**Fig. 1:**
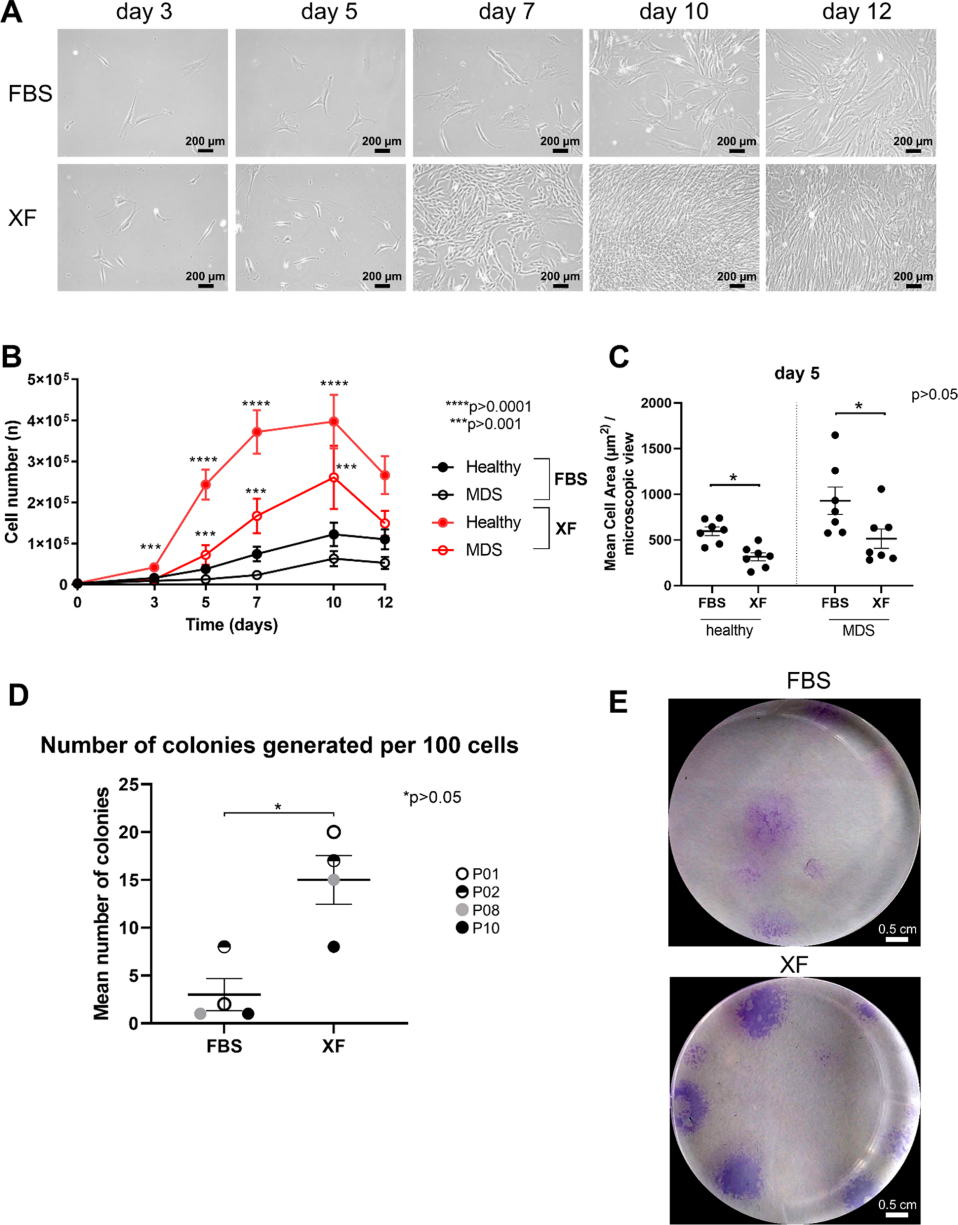
Growth kinetics and clonogenic potential. **A** Exemplary phase contrast images of MSCs from MDS patient P06 cultured with media containing fetal bovine serum (FBS) or xeno-free (XF) medium for 12 days. Cells cultured with XF showed a higher confluence after 7 days. Scale: 200 µm **B** Total cell number obtained upon culturing healthy or MDS MSCs in FBS or XF media. Significantly higher cell numbers were received during expansion with XF compared to FBS for healthy MSCs after 3 days of culture and for MDS MSCs after 5 days of culture (Statistical significance is specified with asterisks ***p ≤ 0.001, ****p ≤ 0.0001). **C** Cell area was measured in representative images from day 5 of culture for *n* = 7 healthy samples and *n* = 7 MDS samples using ImageJ. The graph shows the mean cell area measured per microscopic view. (Statistical significance is specified with asterisks *p ≤ 0.05) **D** Average number of colonies generated per 100 cells at passage 2, by cells assayed in XF or FBS-containing medium (*n* = 4, P01, P02, P08 and P10). (Statistical significance is specified with asterisks *p ≤ 0.05). **D** Representative images of Giemsa staining without magnification showing more stained colonies with XF media compared to FBS

To address the question which medium composition is beneficial for MDS MSC growth, freshly thawed MDS MSCs were cultured with StemMACS human MSC Expansion Media, either containing FBS or XF, for 12 days. Cryo-preserved MSC samples were used from BM aspirations of *n* = 12 patients diagnosed with MDS, and primary BM from young (*n* = 4) or old donors (*n* = 11) after hip joint endoprosthetic surgery served as healthy controls. Cells were previously isolated according to the protocol mentioned in the methods section and stored in passage 1 or 2. During culture in medium containing FBS or XF, cell morphology was observed by phase contrast microscopy. Images of a representative culture series for MDS patient P06 are shown in Fig. 1A. Differences in cell density were observed. To evaluate growth kinetics, cell numbers were counted every 2–3 days. Cell numbers were significantly higher in healthy MSCs after 3 days (FBS: 16 × 10^3^ ± 11 × 10^3^, XF: 42 × 10^3^ ± 24 × 10^3^, ***p = 0.0002) of culture until day 10 (FBS: 122 × 10^3^ ± 112 × 10^3^, XF: 397 × 10^3^ ± 252 × 10^3^, ****p = 0.0001), and in MDS MSCs after 5 days (FBS: 13 × 10^3^ ± 11 × 10^3^, XF: 72 × 10^3^ ± 83 × 10^3^, ***p = 0.0005) of culture until day 10 (FBS: 63 × 10^3^ ± 64 × 10^3^, XF: 261 × 10^3^ ± 268 × 10^3^, ***p = 0.0005) in XF media compared to FBS-containing media (Fig. 1B). MSC cell area was measured in representative microscopic images and showed a decrease during culture with XF medium in healthy and MDS MSCs on day 5 (Fig. 1C). In summary, MSCs from healthy donors as well as from MDS patients cultured in XF medium were smaller and proliferated faster than in serum-containing medium.

### Clonogenic potential of MDS MSCs increases with XF medium

Early data for MSCs showed that they were fibroblastoid colony-forming cells with characteristic features such as adherence to tissue culture plastic and generation of colonies when plated at low densities [33]. The colony-forming efficiency remains an important assay for the quality of cell preparations. The colony forming unit-fibroblast (CFU-F) assay was performed side by side with *n* = 4 MDS samples freshly thawed as described above using 100 cells in triplicates with XF media versus FBS-containing medium. These cells had been frozen in passage 1. Cells assayed in XF media had an average number of CFU-F of 15 ± 5 after 14 days, whereas the cells grown with FBS had 3 ± 3 (Fig. 1D). The number of colonies was significantly higher with the cells assayed in XF compared to the cells in FBS-containing media. Representative images after Giemsa staining of CFU-Fs are shown in Fig. 1E.

### Differentiation capacity of MDS MSCs remains stable with XF medium

**Fig. 2:**
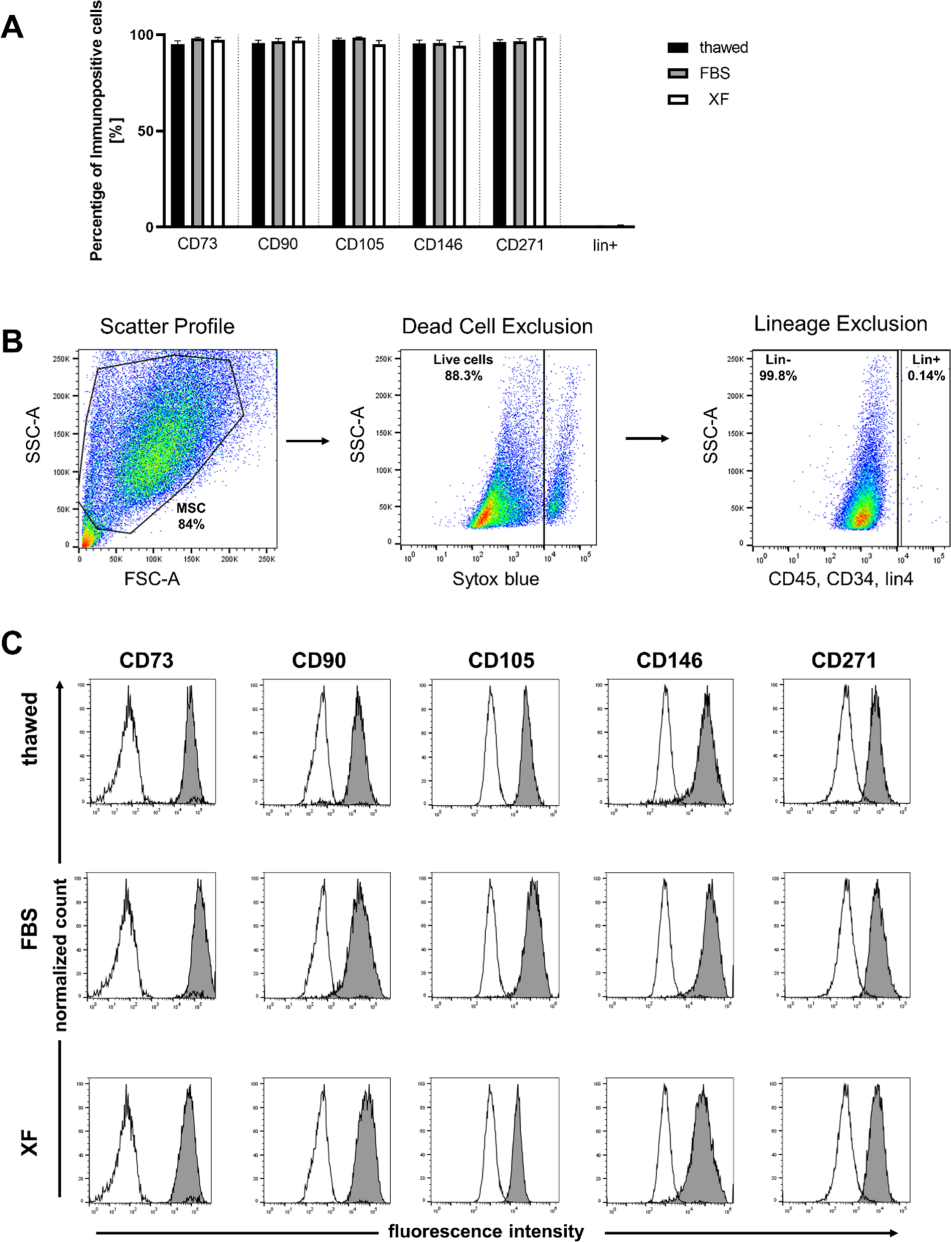
Immunophenotypic characterization. **A** Surface marker expression on cells freshly thawed or expanded over 2 passages with FBS or XF media. The cells are negative for hematopoietic lineage (lin) markers (CD45, CD34, CD2, CD3, CD4, CD7, CD8, CD10, CD11b, CD14, CD19, CD20, CD56, CD235a) and positive for mesenchymal markers (CD73, CD90, CD105, CD146 and CD271) **B** Flow cytometry plots showing the gating strategy with dead cell and lineage exclusion. **C** Representative histograms of thawed or expanded MSC stained for mesenchymal markers CD73, CD90, CD105, CD146 and CD271 (gray histograms). White histograms showing the respective unstained control

**Fig. 3:**
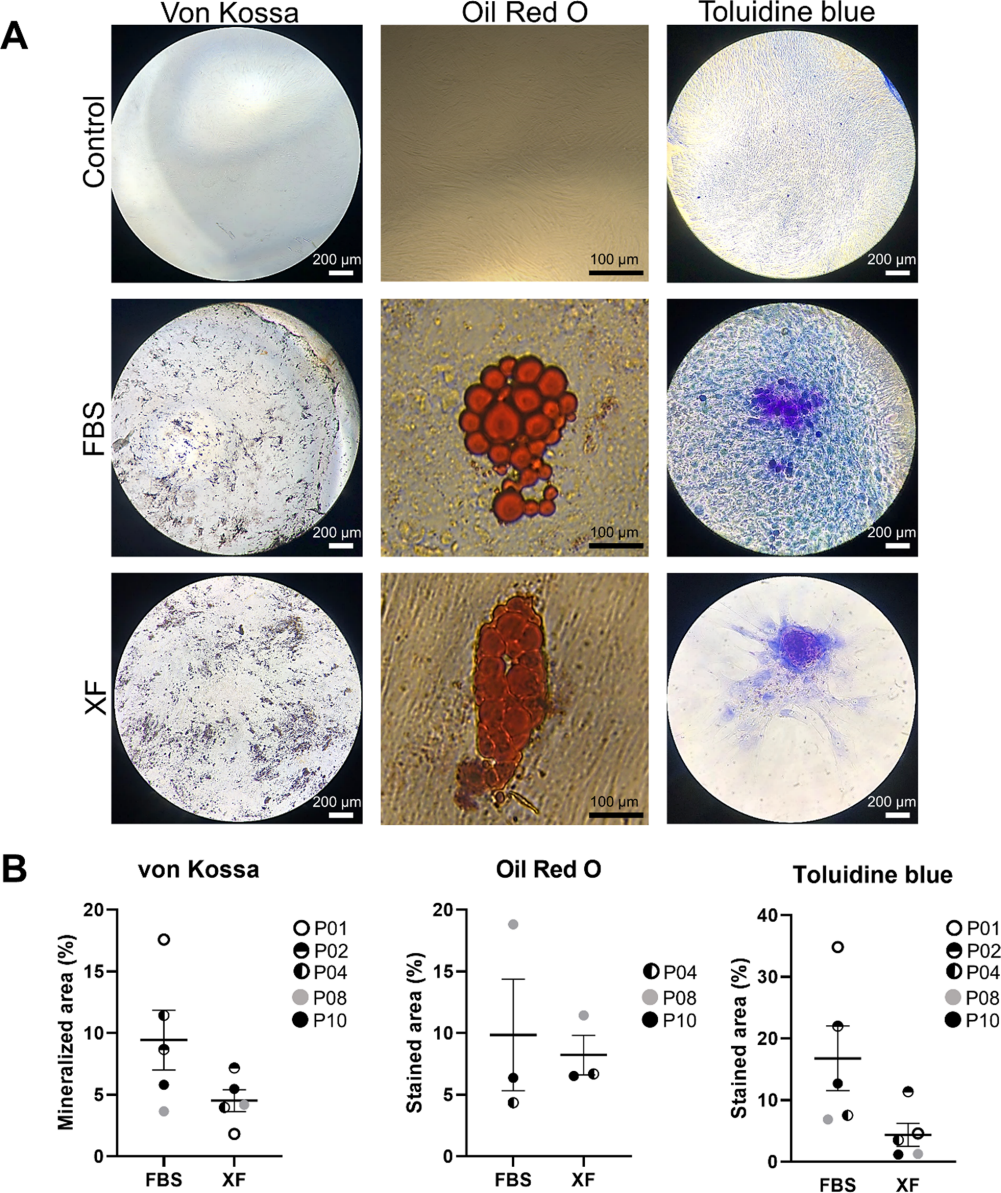
Multilineage differentiation of MDS MSCs. MDS MSCs expanded using XF or FBS-containing media were harvested and plated in respective differentiation media. MDS MCSs differentiated in vitro into osteoblasts (von Kossa, Scale: 200 µm), adipocytes (Oil Red O, Scale: 100 µm) and chondroblasts (Toluidine blue, Scale: 200 µm) exemplary images shown in (**A**) and quantification of the mineralized or stained area in (**B**)

After thawing and expansion over two passages in medium containing FBS or XF, immunophenotypic analysis was performed by flow cytometry for *n* = 9 MDS MSC samples using MSC and hematopoiesis-specific antibodies. Freshly thawed and cultured cells showed similar expression profiles being negative for hematopoietic markers (CD45, CD34, CD2, CD3, CD4, CD7, CD8, CD10, CD11b, CD14, CD19, CD20, CD56, CD235a) (= lineage negative) and positive for the mesenchymal markers CD73, CD90, CD105, CD146 and CD271 (Fig. 2A). Representative Scatter Profile, dead cell exclusion and linage exclusion are shown in Fig. 2B. Representative histograms of the positive markers CD73, CD90, CD105, CD146 and CD271 revealed that most of the cells were mesenchymal (Fig. 2C). The XF medium used in this study did not alter any of the commonly analyzed MSC-associated markers significantly when compared to standard conditions. Furthermore, these cells showed multilineage differentiation capacity along osteogenic, adipogenic and chondrogenic lineages after expansion with FBS or XF for two passages (Fig. 3). Differentiation capacity was assessed for 5 MDS samples and showed comparable results (P01, P02, P04, P08, and P10). After 21 days of differentiation in osteogenic medium, cultured cells showed typical cuboidal and flattened osteoblastic morphology, and matrix mineralization was confirmed by von Kossa staining (Fig. 3A left column). For quantification, the mineralized area was evaluated with ImageJ in MDS MSC samples (*n* = 5). XF-cultured MSCs tended to show lower mineralized areas as compared to FBS-cultured cells, albeit, not reaching statistical significance.(FBS: 9% ± 5%, XF: 5% ± 2%, Fig. 3B left). Adipogenic differentiation of the cells was detected by staining of accumulated lipid vesicles using Oil Red O stain as shown in Fig. 3A middle column. The Oil Red O stained area was quantified for 3 MDS MSC samples, for 2 samples no adipocyte differentiation occurred. Quantification of the percentage of the stained area showed no influence of the culturing condition (FBS: 10% ± 8%, XF: 8% ± 3%, Fig. 3B Oil Red O). Chondrogenic differentiation of MSCs in 3D spheroid culture for 21 days resulted in formation of cartilage tissue containing proteoglycan and was verified by toluidine blue staining (Fig. 3A last column). The Toluidine blue stained area was quantified for 5 MDS MSC samples and showed slightly lower values in XF-cultured MSCs as compared to MSC cultured in FBS. However, this did not reach statistical significance (FBS: 17% ± 12%, XF: 4% ± 4%, Fig. 3B Toluidine blue). In summary, we observed slightly lower rates of osteogenic and chondrogenic differentiation in XF cultured MSCs, which could possibly be explained by the overall smaller cell sizes reached with this culture condition (Fig. 1C) Nevertheless, these results indicate that beside the increased proliferation of MSCs in XF media the expanded cells still keep their MSC functionality.

### “Stemness” gene expression signature is retained in MDS-derived MSCs when cultured with XF medium

**Fig. 4:**
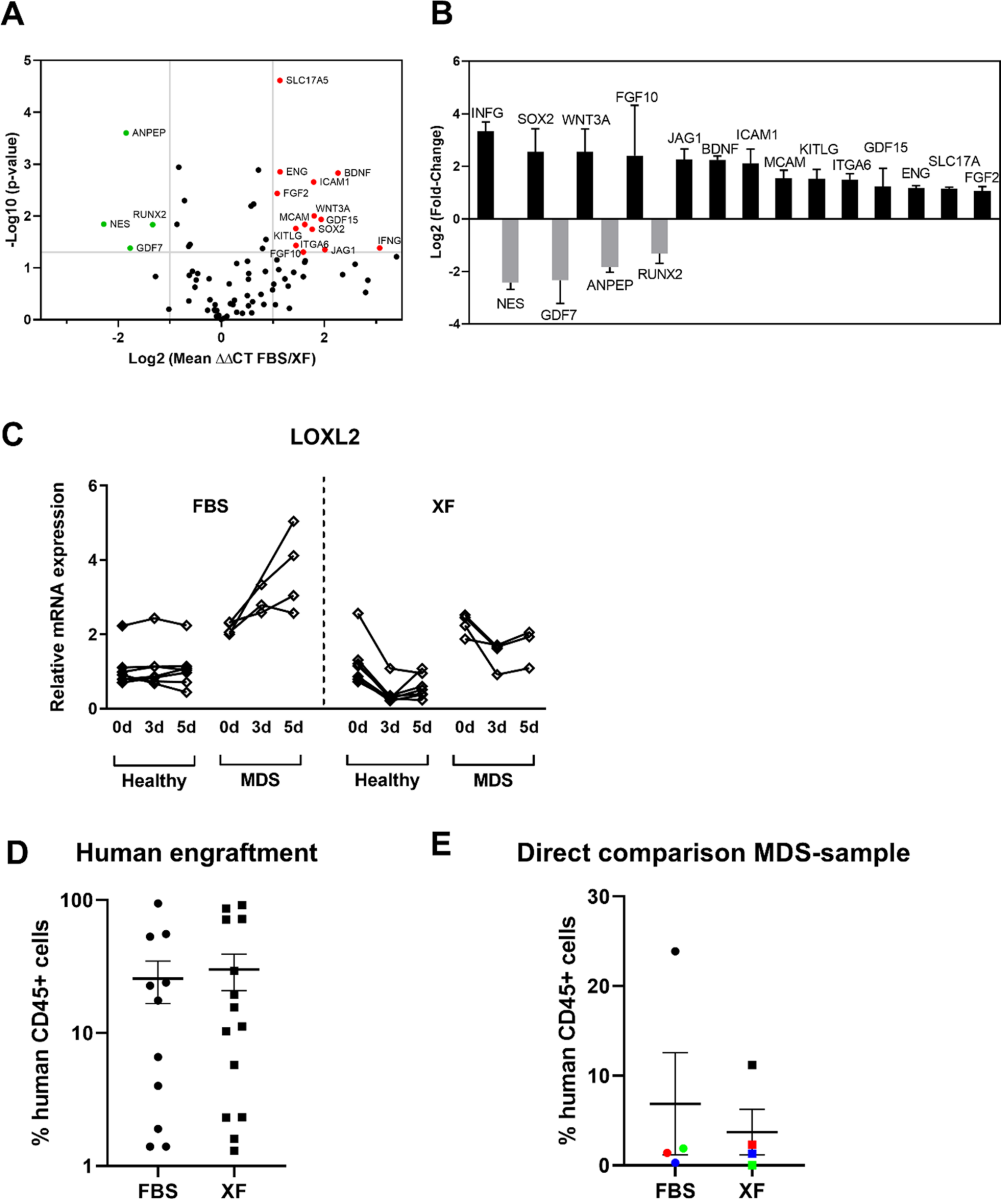
Impact of culture conditions on gene expression and engraftment. **A** Volcano plot shows the expression profile of 82 genes expressed after culture with XF compared to FBS media using the Human Mesenchymal Stem Cell RT^2^ Profiler PCR Array (PAHS-082, SABiosciences, Qiagen, Hilden, Germany). The genes with fold changes higher than the cut-off value (fold change > 2 with *p* < 0.05) were selected. **B** Fourteen transcripts (INFG, SOX2, WNT3A, FGF10, JAG1, BDNF, ICAM1, MCAM, KITLG, ITGA6, GDF15, ENG, SLC17A5 and FGF-2) were upregulated during culture in XF media and four transcripts (NES, GDF7, ANPEP and RUNX2) were downregulated. **C** Relative mRNA expression of the gene LOXL2, a gene our previous data showed upregulation in MDS. **D** Comparison of the mean hCD45 + cell engraftment from MDS patient material co-transplanted with MSCs expanded with FBS or XF media. There was no difference in human engraftment even for the samples from the same patient and time point (**E**)

To assess MSC-specific or associated genes, a RT^2^ Profiler PCR Array was used to evaluate the expression of 84 specific genes related to stemness, MSCs and cell differentiation (Additional file 1: Table S1). MSCs from four different patients were used for analysis after five days of culture with medium containing FBS or XF. Figure 4A shows the expression profile of 82 genes expressed after culture with XF compared to FBS-containing medium. ZFP42 and HNF1A were not detectable. Genes with fold changes higher than the cut-off value (fold change > 2 with *p* < 0.05) were selected. Fourteen transcripts (INFG, SOX2, WNT3A, FGF10, JAG1, BDNF, ICAM1, MCAM, KITLG, ITGA6, GDF15, ENG, SLC17A5 and FGF-2) were upregulated in MDS patient-derived MSCs during culture in XF media and four transcripts (NES, GDF7, ANPEP and RUNX2) were downregulated (Fig. 4B). Together these data showed that cultured MSCs with FBS or XF express MSC specific genes, but their expression pattern is different in dependency of the culture medium. The majority of the upregulated genes in XF culture were associated with a stemness-like profile such as FGF2, WNT3A, SOX2, SLC17A5, BDNF, ICAM1, KITLG and IFNG suggesting expansion of MSCs with XF medium without loss of stemness during proliferation. Furthermore, the evaluation of the gene LOXL2, a gene, our group identified as candidate gene overexpressed in MDS MSCs as compared to healthy MSCs [19], showed dynamic upregulation in FBS-containing medium in a time course experiment, while it maintained a more stable expression on elevated levels in XF medium (Fig. 4C). Our results therefore suggested that XF culture may possibly be preferential for the investigation of MDS-related genes because it keeps gene expression levels more constant as compared to FBS-containing medium.

### MSCs cultured in XF medium enable robust MDS engraftment in immunodeficient mice

We had previously established an MDS xenograft model, which relied on a co-transplantation protocol of patient-derived MSCs, which supported the engraftment of the corresponding HSPCs in immunodeficient mice [19]. In order to evaluate whether different culture conditions of MSCs had any impact on the xenografting performance of MDS patient-derived HSPCs transplanted with this protocol we compared engraftment data generated in experiments using FBS-cultured MSCs versus XF-cultured MSCs. To this end, we were able to compare engraftment data from *n* = 11 MDS patients transplanted with FBS cultured MSCs (previously unpublished) with *n* = 14 MDS patient samples transplanted with XF medium cultured MSCs (*n* = 9 previously published in Schmitt et al. [30], *n* = 5 previously unpublished), patient characteristics are summarized in Table 2. The mean percentages of hCD45 + cells of all mice (human engraftment) were compared between xenografts with human engraftment over 1%. Thereby, no significant difference could be observed between these two groups (FBS: 26% ± 30%, XF: 30% ± 34%, Fig. 4D). Furthermore, from four patients, MSCs from the same time point were transplanted after expansion with FBS or XF. No significant difference was observed between the culture conditions and the human engraftment (FBS: 7% ± 11%, XF: 4% ± 5%, Fig. 4E). Thus, MSCs expanded with XF medium are similarly supportive for MDS HSPC engraftment as those expanded with FBS.

**Table 2:** Number of MDS patients and patient characteristics of the material used for xenotransplantation

Patient characteristics	MSCs expanded with FBS	MSCs expanded with XF
Age	52–83	49–86
IPSS		
low	1	4
int-1	6	5
int-2	4	4
high	0	1
**WHO 2016**		
MDS-MLD	2	3
MDS-RS-MLD	1	1
MDS-EB1	2	3
MDS-EB2	4	3
tMDS	1	2
MDS-U		1
MDS with isolated del(5q)	1	1

The table displays the number of patients used for xenografting and their patient characteristics like age, IPSS and WHO 2016 classification. Engraftment data generated in experiments using FBS cultured MSCs versus XF cultured MSCs were compared. To this end, we were able to compare engraftment data from *n* = 11 MDS patients transplanted with FBS cultured MSCs with *n* = 14 MDS patient samples transplanted with XF medium

*MDS* Myelodysplastic Neoplasms, *IPSS* MDS risk score according to international prognostic scoring system, *FBS* Fetal bovine serum, *int* Intermediate

## Discussion

MSCs isolated from patients with MDS are frequently used in MDS PDX and in vitro experiments [19, 20]. The need for transplanting appropriate numbers of MSCs into mice to generate PDXs requires extensive prior in vitro cell propagation usually utilizing FBS as the main source of growth supplement. However, several studies reported advantages in substitution of FBS by XF medium for MSC large-scale expansion from healthy donors [3, 5, 26]. For example, it can accelerate the production of MSCs [2]. In terms of 3Rs and ethical issues using FBS the goal of the current study is to improve MSC expansion from MDS patient bone marrow with XF culture conditions.

We found that XF medium significantly enhanced proliferation of healthy and MDS MSCs compared to FBS (Fig. 1). These results are in line with previous studies demonstrating that XF could augment the proliferation of healthy bone marrow-derived MSCs or adipose tissue-derived MSCs [2, 34]. Our current study shows that this is also applicable for MSCs derived from patient with myeloid malignancies. Furthermore, the MDS MSCs cultured in XF medium were smaller and able to form more CFU-Fs than after culture in medium containing FBS suggesting that XF medium enhances clonogenic potential and proliferation. This is in line with a previous report showing that MSCs are a heterogenic cell population with some small round cells having a greater potential for multipotential differentiation and self-renewal [35]. Interestingly, results in this study were obtained despite of initial expansion and freezing of all MSCs in medium containing FBS. Before the comparative experiments, all cells were washed twice with the target test medium before seeding in XF or FBS-containing medium, suggesting that possible trace leftovers from FBS had no influence on the effect of XF medium on proliferation and cell morphology.

Beside the advantage of gaining more MSCs from a single MDS patient bone marrow by cultivation with XF medium, the replacement of FBS seems favorable due to minimization of batch-to-batch differences and the unknown composition of FBS. Furthermore, the replacement of FBS reduces the need for animals as a source for the production. For clinical applications, there are concerns about the use of FBS because it may lead to the introduction of xenogeneic antigens with MSC transplant and host immune reaction [36, 37], and possible contamination with non-human pathogens and endotoxins.

To investigate whether the MDS MSCs expanded with XF medium have the same potential as cells expanded with FBS, we assessed for immunophenotypical markers, characteristic abilities of MSCs such as their clonogenic potential and the ability to differentiate into osteoblasts, adipocytes and chondroblasts (Figs. 2 and 3). Our data suggested that MDS MSC culture with XF for two passages supports the proliferation of MSCs. The XF expanded cells exhibited an immunophenotype similar to thawed or cells expanded with FBS and are able to differentiate into osteoblasts, adipocytes and chondroblasts with slightly lower rates of osteogenic and chondrogenic differentiation in XF cultured MSCs, which could possibly be explained by the overall smaller cell sizes reached with this culture condition. These results are in line with reports showing that XF medium can be used for large-scale expansion of adipose tissue-derived MSCs [5] or Whartons Jelly derived MSCs [3]. The differentiation characteristics of MSC after XF medium were more consistent than with FBS medium. This could be due to the fact that for appropriate differentiation experiments, a cell number of at least one million cells are required. For some samples, this was difficult to achieve and took more time with the cells growing in FBS-containing medium. We were able to show that the MSCs expanded with XF medium had higher gene expression of “stemness” markers and a more stable gene expression of candidate genes MDS-related genes such as LOXL2 [19] of the timecourse of culturing (Fig. 4A–C). These findings are congruent with data by Bieback et al. and Lange et al. showing defined genes clustered in ‘‘differentiation/development’’ and ‘‘cell adhesion/extracellular matrix–receptor interaction’’ upregulated in adipose tissue-derived MSCs or BM-MSCs in FBS compared to human platelet lysate. Based on this, we suggest that cells cultivated in human serum reflect more premature stages and less pre-differentiated cells [38, 39]. This is also supported by Dahl et al., who demonstrated that BM-MSCs cultivated in human serum maintained a higher number of genes in unmethylated stages [40].

Taken together, this study offers a detailed characterization of MDS MSCs after expansion under XF conditions and provides an improvement of ex vivo expansion of MDS patient-derived MSCs. With this method, more MDS PDX xenografts can be generated from one sample, enabling more research with better quality toward better or novel therapy options for MDS patients.

## Conclusion

In summary, our results indicate that XF media do not alter typical MSC characteristics. However, the proliferation rate is higher when compared with conventional medium formulations containing bovine serum. Furthermore, the presence of FBS seems to alter MDS-related gene expression during culture and may have an impact on experimental readouts and data interpretation. Our data lead to the conclusion that more MDS MSCs could be generated from single human bone marrow aspirates with XF medium, and that those cells more stable maintain their stem cell potential and gene expression profile. Thus, the protocol for patient-derived MSC expansion should be changed in order to establish more MDS PDX xenografts to improve research on the pathomechanisms of this disease and to find new therapeutic targets.

## Supplementary Information

**Additional file 1 **Other Mesenchymal stem cell genes.

## Data Availability

The datasets used and/or analyzed during the current study are available from the corresponding author on reasonable request.

## Abbreviations

MDS: Myelodysplastic Neoplasms
HSPCs: Hematopoietic stem and progenitor cells
BM: Bone marrow
MSCs: Mesenchymal stroma/stem cells
XF: Xeno-free
FBS: Fetal bovine serum
UBC: Umbilical cord blood
ISCT: International Society for Cellular Therapy
IPSS: International Prognostic Scoring System
WPSS: WHO prognostic scoring system
PDX: Patient-derived xenografts
CFU-Fs: Colony-forming unit fibroblast
MNCs: Mononuclear cells
*α*-MEM: Alpha-minimum essential media
IBMX: 3-Isobutyl-1-methylxanthine
PCR: Polymerase chain reaction
RNA: Ribonucleic acid
SD: Standard deviation
ns: Not significant
CD: Cluster of differentiation
